# Hemostasis during low molecular weight heparin anticoagulation for continuous venovenous hemofiltration: a randomized cross-over trial comparing two hemofiltration rates

**DOI:** 10.1186/cc8191

**Published:** 2009-12-03

**Authors:** Heleen M Oudemans-van Straaten, Muriel van Schilfgaarde, Pascal J Molenaar, Jos PJ Wester, Anja Leyte

**Affiliations:** 1 Department of Intensive Care Medicine, Onze Lieve Vrouwe Gasthuis, Oosterpark 9, 1091 AC Amsterdam, The Netherlands; 2 Department of Clinical Chemistry, Onze Lieve Vrouwe Gasthuis, Oosterpark 9, 1091 AC Amsterdam, The Netherlands; 3 Institutional address: Onze Lieve Vrouwe Gasthuis, PO Box 95500, 1091 AC Amsterdam, The Netherlands

## Abstract

**Introduction:**

Renal insufficiency increases the half-life of low molecular weight heparins (LMWHs). Whether continuous venovenous hemofiltration (CVVH) removes LMWHs is unsettled. We studied hemostasis during nadroparin anticoagulation for CVVH, and explored the implication of the endogenous thrombin potential (ETP).

**Methods:**

This cross-over study, performed in a 20-bed teaching hospital ICU, randomized non-surgical patients with acute kidney injury requiring nadroparin for CVVH to compare hemostasis between two doses of CVVH: filtrate flow was initiated at 4 L/h and converted to 2 L/h after 60 min in group 1, and *vice versa *in group 2. Patients received nadroparin 2850 IU i.v., followed by 380 IU/h continuously in the extracorporeal circuit. After baseline sampling, ultrafiltrate, arterial (art) and postfilter (PF) blood was taken for hemostatic markers after 1 h, and 15 min, 6 h, 12 h and 24 h after converting filtrate flow. We compared randomized groups, and 'early circuit clotting' to 'normal circuit life' groups.

**Results:**

Fourteen patients were randomized, seven to each group. Despite randomization, group 1 had higher SOFA scores (median 14 (IQR 11-15) versus 9 (IQR 5-9), p = 0.004). Anti-Xa _art _activity peaked upon nadroparin bolus and declined thereafter (p = 0.05). Anti-Xa _PF _did not change in time. Anti-Xa activity was not detected in ultrafiltrate. Medians of all anti-Xa samples were lower in group 1 (anti-Xa _art _0.19 (0.12-0.37) vs. 0.31 (0.23-0.52), p = 0.02; anti-Xa _PF _0.34 (0.25-0.44) vs. 0.51 (0.41-0.76), p = 0.005). After a steep decline, arterial ETP_AUC _tended to increase (p = 0.06), opposite to anti-Xa, while postfilter ETP_AUC _increased (p = 0.001). Median circuit life was 24.5 h (IQR 12-37 h). Patients with 'short circuit life' had longer baseline prothrombin time (PTT), activated thromboplastin time (aPTT), lower ETP, higher thrombin-antithrombin complexes (TAT) and higher SOFA scores; during CVVH, anti-Xa, and platelets were lower; PTT, aPTT, TAT and D-dimers were longer/higher and ETP was slower and depressed.

**Conclusions:**

We found no accumulation and no removal of LMWH activity during CVVH. However, we found that early circuit clotting was associated with more severe organ failure, prior systemic thrombin generation with consumptive coagulopathy, heparin resistance and elevated extracorporeal thrombin generation. ETP integrates these complex effects on the capacity to form thrombin.

**Trial registration:**

Clinicaltrials.gov ID NCT00965328

## Introduction

Acute kidney injury (AKI) is a severe complication of critical illness, generally developing as a component of multiple organ failure. If renal replacement therapy is required, continuous techniques are often preferred especially in patients with instable circulation. To prevent clotting in the extracorporeal circuit, continuous anticoagulation is needed and heparins are the classic choice. Both unfractionated heparin and low molecular weight heparins (LMWHs) are used. LMWHs have the advantage that their pharmacokinetics are more predictable due to less binding to proteins and cells [1]. Their clearance is, however, slower. In addition, renal insufficiency increases half-life of smaller heparin fragments resulting in accumulation of anti-Xa activity, but not of anti-IIa activity [2,3]. Bleeding complications increase when glomerular filtration rate falls below 30 ml/min. The biological activity and behavior of LMWHs during continuous renal replacement therapy is still controversial. Although a previous study found no elimination of LMWHs [4], a recent small study using enoxaparin reported partial removal of anti-Xa activity by filtration and dialysis [5].

Hemostatic changes during continuous renal replacement therapy in the critically ill are complex due to simultaneous pro- and anticoagulant processes. Routine prothrombin time (PTT) and activated partial thromboplastin time (aPTT) assays monitor clot formation but are insensitive to hypercoagulant states, especially during anticoagulation. Plasma anti-Xa activity measures anticoagulant activity of LMWHs. The endogenous thrombin potential (ETP) reflects thrombin generation beyond the initiation of clot formation and may be more informative with regard to the presence of an anti- or procoagulant state [6].

The aim of this explorative study in patients with AKI receiving the LMWH nadroparin for anticoagulation of the continuous venovenous hemofiltration (CVVH) circuit was to determine whether anti-Xa activity accumulates, whether it is removed by filtration, and to determine whether ETP could have a role in monitoring hemostasis and circuit clotting. As heparins are a heterogenic mixture of molecules, drug concentrations cannot be measured directly. We therefore assessed its anticoagulant activity (anti-Xa), which is its clinically relevant effect.

## Materials and methods

### Study design and setting

This prospective randomized cross-over trial was conducted in a 20-bed closed format general intensive care unit (ICU) of a teaching hospital. CVVH is the only renal replacement modality used in the unit and is performed under responsibility of the intensivists. Nadroparin is the standard anticoagulant for CVVH in patients without an increased risk of bleeding. The institutional review board approved the study according to European and Dutch legislation. Written informed consent was acquired from the patient or his legal representative.

### Patients and randomization

Adult critically ill patients with acute renal failure requiring CVVH were eligible for inclusion. Exclusion criteria were (recent) bleeding or a suspicion of bleeding necessitating transfusion, need of therapeutic anticoagulation or (suspected) heparin-induced thrombocytopenia. CVVH was initiated when, after resuscitation of the circulation, oliguria persisted and was accompanied by a steep rise in serum creatinine, or at a non-declining rise in creatinine in non-oliguric patients. Randomization was computer-based. When inclusion and exclusion criteria were checked in the patient data management system (MetaVision^®^, IMDSoft, Tel Aviv, Israel), the system automatically randomized the patients.

### Study protocol

Patients were randomized to one of two groups. In group 1, postdilutional CVVH was initiated at a filtrate flow of 4 L/h (blood flow 220 ml/min), which was converted to 2 L/h (blood flow 150 ml/min) after 60 minutes. In group 2, postdilutional CVVH was initiated at a filtrate flow of 2 L/h and converted to 4 L/h after 60 minutes. The cross-over design was chosen to detect differences in plasma and ultrafiltrate anti-Xa activity in case of elimination of anti-Xa activity by filtration. The 4 L/h dose is our default starting dose in the unit, which is normally reduced to 2 L/h if uremic toxins are low and circulation has stabilized.

We used a 1.9 m^2 ^cellulose triacetate hollow fiber membrane (UF 205, Nipro, Osaka, Japan), bicarbonate buffered replacement fluids heated to 39°C, and the Aquarius device (Edwards LifeSciences, S.A., Saint-Prex, Switzerland). Nadroparin (Sanofi-Synthelabo, Maassluis, the Netherlands) was added to the one-liter priming solution (2850 IU). Patients received an intravenous bolus of 2850 IU nadroparin at initiation of CVVH, or 3800 IU when body weight exceeded 100 kg, followed by a continuous infusion in the extracorporeal circuit before the filter of 380 or 456 IU/h, respectively.

After baseline sampling of arterial blood, samples of ultrafiltrate, arterial blood and postfilter blood were taken one hour after the start of CVVH, and at 15 minutes, 6 hours, 12 hours and 24 hours after the conversion from 4 to 2 L/h or from 2 to 4 L/h to measure antithrombin (at baseline only), anti-Xa activity, PTT, aPTT, platelet count, ETP, prothrombin fragments 1 and 2 (F1+2), thrombin-antithrombin complexes (TAT) and D-dimers. Postfilter samples were taken directly after the filter, before infusion of the replacement fluid. Results of postfilter measurements are actual values, not corrected for hemoconcentration, unless indicated differently. Circuits were disconnected at high prefilter or transmembrane pressure (both more than 300 mmHg), if vascular access failed, routinely after 72 hours or for clinical reasons (renal recovery, transport). Before initiation of CVVH, patients received once daily subcutaneous nadroparin for thromboprophylaxis at a dose of 2850 IU or 3800 IU if body weight exceeded 100 kg.

### Biochemical measurements

Blood was collected into a 4.5 ml tube containing 0.105 M sodium citrate for coagulation measurements and in a 4 ml tube containing 7.5% potassium EDTA for hemocytometry (Becton Dickinson, Plymouth, UK). Citrated blood was centrifuged at 1500 g for 10 minutes, and plasma aliquots were stored at -80°C. Aliquots of ultrafiltrate samples were frozen at -80°C until use. The following assays were performed immediately after sampling: PTT (Innovin), aPTT (Actin FS) and antithrombin (Berichrom ATIII) on a Sysmex CA-1500 coagulation analyzer (all Siemens Healthcare Diagnostics, Deerfield, IL, USA), and platelet counts on a Sysmex XE-2100 hematology analyzer (Sysmex, Kobe, Japan).

Anti-Xa activity was determined in ultrafiltrates and citrated plasma to assess the anticoagulant activity of the LMWH nadroparin using the Coamatic Heparin kit (Chromogenix, Instrumentation Laboratory Company, Lexington, MA, USA). For determination of anti-Xa in ultrafiltrate, anti-Xa activity was determined after addition of an equal volume of normal plasma (Standard Human Plasma, Siemens Healthcare Diagnostics Deerfield, IL, USA) to the ultrafiltrate to provide for a suitable matrix and the presence of antithrombin. The sensitivity of our anti-Xa assay, (detection limit 0.01 U/ml) albeit negatively influenced by a factor 2 when measuring ultrafiltrate because of the need to add normal plasma, is sufficient to demonstrate relevant anti-Xa removal. Analytical precision, characterized by a coefficient of variation percentage of less than 2.5 at the higher anti-Xa levels, is adequate to detect relevant accumulation in plasma if present.

The ETP was measured as an overall indicator of hemostasis. The ETP monitors the thrombin-forming capacity of plasma, including the generation and inhibition of thrombin generation beyond the initiation of fibrin clot formation providing an overall assessment of hemostasis and potential extra-hemostatic effects of the generated thrombin [6]. The ETP is characterized by 'lag time' (ETP_Tlag _(s)), 'time to maximal activity' (ETP_Tmax _(s)), 'maximal activity' (ETP_Cmax _(mA/min)) and the main parameter: 'area under the curve' (ETP_AUC _(mA)); the latter represents the total thrombin formation. ETP was determined on the BCS-XP (Siemens Healthcare Diagnostics, Deerfield, IL, USA) using the ETP-B protocol and reagents as provided and described by the manufacturer. In this protocol, thrombin formation is triggered via the addition of Innovin up to a final concentration of 300 pM tissue factor, also providing for phospholipids. We have established a provisional reference range in our laboratory in 20 adults, representing +/- three standard deviations from the mean ETP_Tlag _14.4 to 22.1 s, ETO_Tmax _48.5 to 60.0 s, ETP_Cmax _115 to 148 mA/min, and ETP_AUC _346 to 520 mA.

Coagulation activation was additionally assayed by measuring the concentration of F1+2 and TAT. F1+2 are specifically generated during the conversion of prothrombin to thrombin. F1+2 levels were determined using the Enzygnost F1+2 (monoclonal) ELISA kit (Siemens Healthcare Diagnostics, Deerfield, IL, USA). Normal F1+2 values are reported to range from 69 to 229 pmol/l (kit insert information as provided by the manufacturer).

Once thrombin is generated one of the mechanisms of the body to down-regulate thrombin is to form TAT. TAT therefore reflects combined pro- and anticoagulant activity. TAT was determined using the Enzygnost TAT micro test kit. Normal values are reported to range from 1 to 4.1 μg/l (kit insert information). D-dimers are early fibrin degradation products, and therefore markers of recent thrombus formation. D-dimer concentrations were determined using the Tina-quant assay (Roche Diagnostics, Indianapolis, In, USA). Normal values are less than 0.5 μg/ml (kit insert information).

### Clinical measurements

Severity of illness was scored using the Acute Physiology and Chronic Health Evaluation (APACHE) II and III systems and the Simplified Acute Physiology Score (SAPS) II system over the first 24 hours of ICU admission. The Sequential Organ Failure Assessment (SOFA) score as defined by the Dutch National Intensive Care Evaluation [7] was taken at the start of CVVH [8-11]. Renal function was classified according to the RIFLE (Risk, Injury, Failure) System [12]. Risk was scored as 1, injury as 2 and failure as 3.

### Data analysis

In this explorative study, the data are analyzed for the randomized groups separately, for the entire group of patients, for patients with early circuit clotting compared with those with normal circuit life and for patients with high and low SOFA score separately. 'Early circuit clotting' was defined a circuit life less than the lower quartile, high SOFA score as SOFA score higher than the median. The data are presented as medians (interquartile ranges (IQR)). We used the Friedman test to detect changes of a variable in time, the Mann-Whitney U test (asymptomatic two-tailed) to compare samples between groups, the Wilcoxon Signed Rank test to compare paired samples and the Spearman rank correlation coefficient (two-tailed) to determine whether variables were related. A *P*-value less than 0.05 was considered statistically significant. Because of the explorative nature of the study we did not correct for multiple testing. We used SPSS 17.0 (SPSS Inc., Chicago, IL, USA) for analysis.

## Results

Fourteen medical patients were included in this study; seven were randomized to the 4 L to 2 L group (group 1) and seven to the 2 L to 4 L group (group 2). Baseline patient characteristics are presented in Table 1. Despite randomization, patients in group 1 were more severely ill. The difference was significant for the SOFA score at start CVVH (*P *= 0.004). During the study period, four patients received a red blood cell transfusion, two in each group, none of the patients received plasma or platelet transfusion.

**Table 1:** Patient characteristics

	Group 1 4 L to 2 L/h		Group 2 2 L to 4 L/h		
	n = 7		N = 7		
Age (years)	65	(38-69)	65	(60-75)	
Male/female (n)	5/2		5/2		
Body weight (kg)	75	(72-80)	75	(60-98)	
Cause of ARF (n)					
sepsis	5		5		
cardiogenic shock	2		1		
liver failure			1		
APACHE II	35	(29-38)	25	(19-31) 0.07	
APACHE II _predicted mortality _(%)	85	(72-88)	56	(21-73) 0.10	
APACHE III	128	(104-138)	94	(50-148)	0.18
SOFA start CVVH	14	(11-15)	9	(5-9)	0.004
Hemoglobin (mmol/L)	5.7	(4.8-6.5)	5.8	(5.6-6.1)	
Hematocrit	0.29	(0.25-0.30)	0.28	(0.26-0.30)	
PTT (sec)	11.8	(10.8-14.5)	11.4	(11.1-12.7)	0.95
aPTT (sec)	32.3	(25.5-36.7)	28.2	(20.3-41.7)	0.57
Antithrombin (%)	79	(43-86)	46	(17-64)	0.23
Platelet count (10^9^/L)	182	(163-286)	139	(96-221)	0.26
Anti-Xa activity (IU)	0.11	(0.00-0.55)	0.01	(0.00-0.15)	0.30
ETP_Tlag _(sec)	23.0	(18.6-31.0)	19.7	(14.3-22.1)	0.13
ETP_Tmax _(sec)	53.1	(46.0-81.4)	52.7	(46.1-54.9)	0.80
ETP_Cmax _(mA/min)	108	(91-125)	114	(75-153)	0.48
ETP_AUC _(mA)	275	(137-379)	341	(223-401)	0.54
F1+2 (pmol/L)	313	(158-689)	216	(156-288)	0.32
TAT (μg/L)	20.7	(4.8-28.9)	8.3	(6.7-10.5)	0.18
D-dimers (μg/ml)	14.2	(7.6-43.8)	4.1	(1.5-29.8)	0.25
Creatinine (μmol/L)	297	(221-453)	251	(141-329)	0.54
Urea (mmol/L)	34	(15-49)	36	(10-47)	0.81
RIFLE score	3		3		1.0

Values in median (interquartile range) unless indicated differently.

APACHE = Acute Physiology and Chronic Health Evaluation; aPTT = activated thromboplastin time; CVVH = continuous venovenous hemofiltration; ETP = endogenous thrombin potential; ETP_AUC _= area under the curve of the thrombin generation curve; ETP_Cmax _= maximal activity of ETP; ETP_Tlag _= ETP lag time = ETP_Tmax _= time to max ETP activity; F1+2 = prothrombin fragment; PTT = prothrombin time; SOFA = Sequential Organ Failure Assessment; TAT = thrombin-antithrombin complexes.

RIFLE = Risk = 1, Injury = 2, Failure = 3.

### Coagulation markers in randomized groups

**Figure 1:**
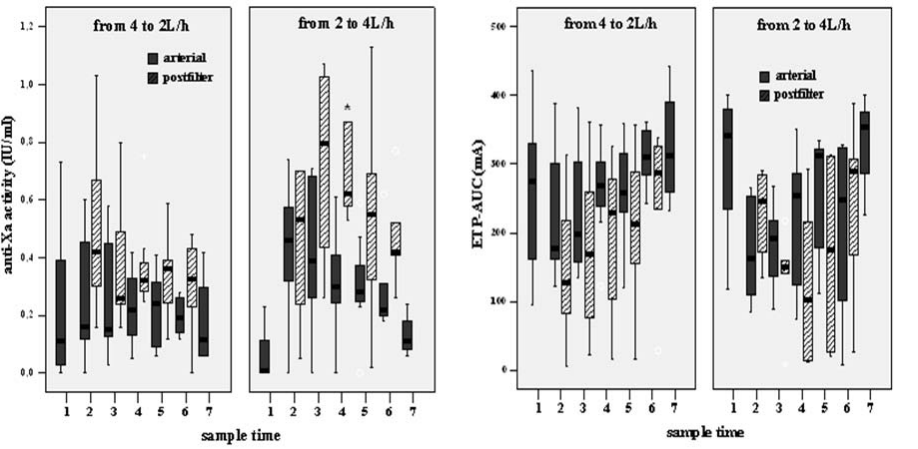
Arterial and postfilter anti-Xa activity and ETP_AUC _are presented for the two randomized groups. Sample time 1 = baseline; sample time 2 = 60 minutes after start continuous venovenous hemofiltration; samples time 3 = 15 minutes after changing filtrate rate; samples time 4 = 6 hours after changing filtrate rate; samples time 5 = 12 hours after changing filtrate rate; samples time 6 = 24 hours after changing filtrate rate; sample time 7 = 4 hours after discontinuation of continuous venovenous hemofiltration). ETP_AUC _= area under the curve of the endogenous thrombin potential. * significantly different between groups.

The course of arterial and postfilter anti-Xa and ETP_AUC _is presented for the two randomized groups separately in Figure 1. Median anti-Xa of all samples during CVVH was significantly lower in group 1 than in group 2, both in arterial blood and in postfilter blood (Table 2). Anti-Xa activity was not detectable in the ultrafiltrate. Median ETP_AUC _during CVVH, was higher in group 1, while postfilter ETP_AUC _values were not significantly different (Table 2). Ranges were large. ETP activity was not detected in ultrafiltrate.

**Table 2:** Comparison of markers of coagulation during CVVH in arterial and postfilter blood between randomized groups

during CVVH and nadroparin infusion (arterial)				during CVVH (postfilter)		
	**Group 1** **4 to 2 L/h**	**Group 2** **2 to 4 L/h**	***P *value**	**Group 1** **4 to 2 L/h**	**Group 2** **2 to 4 L/h**	***P *value**

anti-Xa (IU/ml)	0.19 (0.12-0.37)	0.31 (0.23-0.52)	0.02	0.34 (0.25-0.44)	0.51 (0.41-0.76)	0.005

PTT (sec)	11.1 (10.8-12.6)	11.5 (11.1-12.8)	0.30	10.7 (10.2-12.0)	11.1 (10.4-11.8)	0.80

aPTT (sec)	28.0 (25.4-33.3)	29.3 (22.1-40.5)	0.95	27.0 (24.5-30.7)	28.3 (22.2-35.2)	0.80

platelets (10^9^/l)	164 (131-242)	129 (114-210)	0.05	200 (161-276)	172 (140-274)	0.23

ETP_Tlag _(sec)	18.5 (16.7-23.6)	20.9 (15.1-23.2)	0.84	18.1 (15.5-23.4)	17.8 (12.9-22.3)	0.30

ETP_Tmax _(sec)	47.2 (41.5-56.6)	45.8 (40.9-55.0)	0.93	43.9 (34.8-52.0)	40.1 (35.0-43.8)	0.19

ETP_Cmax _(mA/min)	113 (96-141)	115 (83-134)	0.25	127 (100-149)	143 (99-166)	0.21

ETP_AUC _(mA)	280 (175-338)	209 (109-209)	0.03	212 (99-309)	189 (38-289)	0.45

F1+2 (pmol/L)	298 (198-482)	228 (159-332)	0.06	456 (306-787)	320 (188-455)	0.008

TAT (μg/L)	9.3 (6.7-23)	5.5 (4.8-9.6)	0.001	20 (9.2-53.8)	8.3 (6.2-17.6)	0.001

D-dimers (μg/ml)	11.1 (7.8-29.5)	3.8 (2.0-24.8)	0.03	17.8 (11.0-43.2)	5.7 (3.1-41.8)	0.14

Values are medians (interquartile range) of all samples. Postfilter values are actual values, not corrected for hemoconcentration

aPTT = activated thromboplastin time; CVVH = continuous venovenous hemofiltration; ETP = endogenous thrombin potential; ETP_AUC _= area under the curve of the thrombin generation curve; ETP_Cmax _= maximal activity of ETP; ETP_Tlag _= ETP lag time = ETP_Tmax _= time to max ETP activity; F1+2 = prothrombin fragment; PTT = prothrombin time; TAT = thrombin-antithrombin complexes.

In patients of group 1, median values of F1+2 and TAT were (or tended to be) higher in group 1 than in group 2. Arterial D-dimers were higher in group 1, while postfilter D-dimers were not significantly different between groups (Table 2).

Differences remained after correction for different degrees of hemoconcentration in postfilter blood (0.70 at 4 L/h and 0.78 at 2 L/h).

### Anti-Xa and ETP activity in all patients

**Figure 2:**
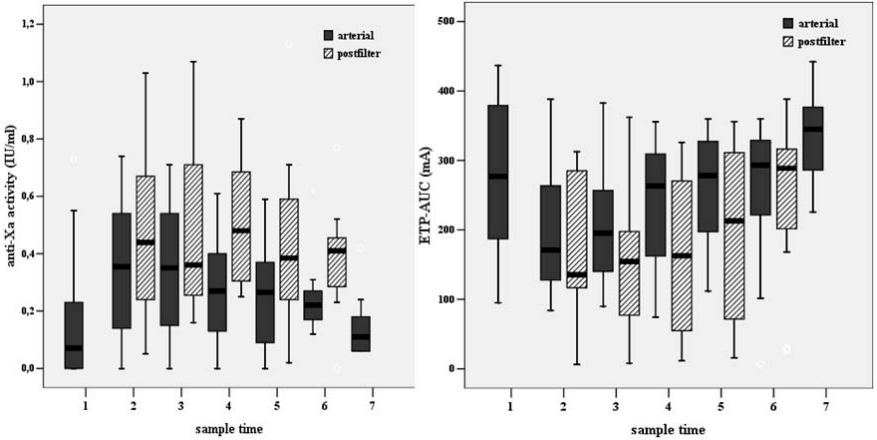
**Arterial and postfilter anti-Xa activity and ETP_AUC _for all patients**. ETP_AUC _= area under the curve of the endogenous thrombin potential.

Arterial anti-Xa activity peaked upon the administration of the intravenous bolus of nadroparin, followed by a gradual decline during the course of CVVH (*P *= 0.05). Postfilter anti-Xa did not significantly change in time. Postfilter anti-Xa activity was significantly higher than arterial anti-Xa with a median ratio of 1.7 (IQR 1.4 to 2.1; Figure 2).

The course of arterial ETP_AUC _was opposite to anti-Xa activity with lowest value after the nadroparin bolus. During CVVH, arterial ETP_AUC _tended to increase again (*P *= 0.06), whereas postfilter ETP_AUC _significantly increased in time (*P *= 0.001). Postfilter ETP_AUC _was significantly lower than arterial ETP_AUC _(Figure 2).

Medians of postfilter F1+2, TAT and D-dimers were significantly higher than arterial values. Postfilter ranges were high.

### Relation between ETP, anti-Xa, other markers of coagulation and severity of organ failure

Median baseline arterial ETP_AUC _was 277 mA (IQR 175 to 385). Baseline ETP_AUC _correlated inversely to PTT (R = -0.80, *P *= 0.001), aPTT (R = -0.69, *P *= 0.006), TAT (R = -0.69, *P *= 0.06) and SOFA score (R = -0.70, *P *= 0.001), but not to anti-Xa, F1+2 and D-dimers. During CVVH and nadroparin infusion, arterial ETP_AUC _correlated inversely to aPTT at all sample times (R = -0.60 to -0.82, *P *= 0.03 to 0.001) and to PTT at t2 and t4 (R = -0.77, *P *= 0.001 and R = -0.64, *P *= 0.01, respectively); postfilter ETP_AUC _did not correlate with aPTT except at t5 (R = -0.65, *P *= 0.02), and not with PTT, anti-Xa, F1+2, TAT and D-dimers.

Arterial anti-Xa at t1 and t2 (one hour after the nadroparin bolus) correlated with antithrombin (R = 0.54, *P *= 0.048 and R = 0.48, *P *= 0.08). Anti-Xa activity was not related to body weight. There was a positive correlation between arterial antithrombin and ETP_Cmax _at t1 and t2 (R = 57, *P *= 0.03 and R = 0.79, *P *= 0.001) and ETP_AUC _at t1 and t2 (R = 0.46, *P *= 0.10 and R = 0.41, *P *= 0.14). ETP and anti-Xa correlated negatively if all samples were taken together (R = -0.36, *P *= 0.001).

### Relation between markers of coagulation, severity of organ failure and circuit life

Median circuit life was 24.5 hours (IQR 12 to 37 hours). Short circuit life was defined as 12 hours of less (the lower quartile). At baseline, patients with short circuit life had a longer PTT, aPTT, higher TAT and lower ETP. They also had higher SOFA scores (Table 3). During CVVH and nadroparin infusion, anti-Xa and platelets were significantly lower in patients with short circuit life, PTT, aPTT, TAT and D-dimers were significantly longer or higher and ETP was slower and depressed (Table 3).

**Table 3:** Comparison of baseline markers of coagulation and severity of organ failure between patients with circuit life of 12 hours or less (lower quartile) and those with circuit life more than 12 hours

Circuit life	≤12 h n = 4	>12 h n = 10	*P *value	≤12 h n = 4	>12 h n = 10	*P *value
**Baseline (arterial)**						
			
antithrombin (%)	40 (17-71)	61 (43-87)	0.20			
			
anti-Xa (IU/ml)	0.12 (0-0.47)	0.07 (0-0.17)	0.94			
			
PTT (sec)	12.6 (12.6-30.5)	11.4 (10.9-11.6)	0.004			
			
aPTT (sec)	66 (33-144)	27 (21-35)	0.02			
			
platelets (10^9^/L)	159 (86-224)	173 (115-280)	0.54			
			
ETP_Tlag _(sec)	27 (15-34)	20 (19-22)	0.20			
			
ETP_Tmax _(sec)	47 (26-65)	54 (50-55)	0.32			
			
ETP_Cmax_	76 (58-112)	120 (105-148)	0.048			
			
ETP_AUC _(mA)	162 (122-256)	349 (241-410)	0.07			
			
F1+2 (pmol/L)	258 (156-651)	237 (149-388)	0.74			
			
TAT (μg/L)	23.0 (18.8-34.9)	7.8 (5.2-13.1)	0.01			
			
D-dimers (μg/ml)	19.4 (4.1-60.8)	8.9 (2.3-25.7)	0.40			
			
SOFA start CVVH	15 (14-15)	9 (8-11)	0.02			

**during CVVH and nadroparin infusion (arterial)**				**during CVVH (postfilter)**		

anti-Xa (IU/ml)	0.13 (0.04-0.39)	0.28 (0.21-0.45)	0.003	0.24 (0.16-0.60)	0.43 (0.32-0.76)	0.003

PTT (sec)	13.7 (11.8-30.6)	11.1 (10.8-11.6)	< 0.001	13.2 (11.6-32.4)	10.5 (10.1-11)	< 0.001

aPTT (sec)	38.4 (31.9-105.5)	27.1 (22.9-31.1)	< 0.001	37.8 (30.0-63.2)	25.9 (23.1-29.4)	< 0.001

platelets (10^9^/L)	135 (114-239)	154 (119-212)	0.55	162 (118-196)	198 (159-283)	0.01

ETP_Tlag _(sec)	22.9 (17.2-36.2)	19.2 (15.9-22.4)	0.03	19.8 (17.2-29.4)	17.4 (12.9-21.5)	0.02

ETP_Tmax _(sec)	55.7 (46.8-77.7)	44.2 (39.8-54.6)	< 0.001	51.0 (45.2-55.0)	38.9 (34.0-44.5)	< 0.001

ETP_Cmax _(mA/min)	89 (48-109)	120 (93-143)	< 0.001	120 (86-128)	143 (111-168)	0.004

ETP_AUC _(mA)	173 (128-280)	258 (167-327)	0.08	173 (122-251)	213 (29-310)	0.71

F1+2 (pmol/L)	192 (148-297)	288 (176-362)	0.81	313 (187-991)	382 (254-515)	0.78

TAT (μg/L)	27 (18.5-77.1)	8.2 (6.6-11.4)	< 0.001	50.9 (21.6-126)	8.9 (7.1-15.2)	< 0.000

D-dimers (μg/ml)	20.2 (12.4-46.4)	7.8 (2.2-22.4)	0.002	36.4 (15.6-59.4)	11.0 (3.3-29.7)	0.002

Values in median (interquartile range). Values during CVVH are medians of all samples. Postfilter values are actual values, not corrected for hemoconcentration.

aPTT = activated thromboplastin time; CVVH = continuous venovenous hemofiltration; ETP = endogenous thrombin potential; ETP_AUC _= area under the curve of the thrombin generation curve; ETP_Cmax _= maximal activity of ETP; ETP_Tlag _= ETP lag time = ETP_Tmax _= time to max ETP activity; F1+2 = prothrombin fragment; PTT = prothrombin time; SOFA = Sequential Organ Failure Assessment; TAT = thrombin-antithrombin complexes.

Median SOFA score was 10. Patients with high SOFA score (>10) had longer PTT, aPTT, a depressed ETP, high TAT and D-dimers and a significantly shorter circuit life. During CVVH anti-Xa was lower and postfilter ETP was slow and depressed (Table 4).

**Table 4:** Comparison of baseline markers of coagulation and circuit life between patients with SOFA score of 10 or less (median) and those with SOFA score of more than 10

SOFA score	≤10 n = 7	>10 n = 7	*P *value	≤10 n = 7	>10 n = 7	*P *value
**Baseline (arterial)**						
			
antithrombin (%)	58 (34-79)	52 (36-86)	0.24			
			
anti-Xa (IU/ml)	0.06 (0.00-0.15)	0.11 (0.00-0.55)	0.94			
			
PTT (sec)	11.4 (11.0-11.5)	12.0 (11.4-24.2)	0.004			
			
aPTT (sec)	24.3 (20.3-35.1)	36.2 (27.5-95.3)	0.02			
			
Platelets (10^9^/L)	143 (96-221)	179 139-267)	0.54			
			
ETP_Tlag _(sec)	19.7 (18.4-22.1)	23.0 (18.6-31.0)	0.24			
			
ETP_Tmax _(sec)	54.8 (51.6-55.7)	48.5 (39.6-54.8)	0.37			
			
ETP_Cmax_	139 (113-153)	97 (61-119)	0.05			
			
ETP_AUC _(mA)	359 (246-437)	187 (118-279)	0.08			
			
F1+2 (pmol/L)	216 (125-288)	314 (165-689)	0.81			
			
TAT (μg/L)	7.4 (5.2-9.0)	20.7 (15.1-29.0)	0.008			
			
D-dimers (μg/ml)	9 (1.5-29.8)	14 (4.3-43.8)	0.47			
			
circuit life (h)	32 (25-39)	12 (4-24)	0.002			

**during CVVH and nadroparin infusion (arterial)**				**during CVVH (postfilter)**		

anti-Xa (IU/ml)	0.28 (0.20-0.45)	0.16 (0.10-0.38)	0.008	0.53 (0.35-0.71)	0.32 (0.24-0.54)	0.003

PTT (sec)	11.3 (11.0-11.8)	11.4 (10.8-14.6)	0.18	10.7 (10.2-11.2)	10.7 (10.3-12.4)	0.10

aPTT (sec)	26 (21.9-32.2)	28.6 (27.1-36.6)	0.001	25.9 (21.7-30.8)	28.7 (25-5.32.5)	0.006

Platelets (10^9^/L)	140 (116-196)	160 (119-251)	0.65	176 (138-217)	193 (154-281))	0.85

ETP_Tlag _(sec)	20.3 (16.7-22.3)	18.8 (15.8-23.8)	0.61	17.1 (13.0-21.3)	18.3 (16.1-24.2)	0.06

ETP_Tmax _(sec)	45.8 (40.4-54.7)	50.8 (42.8-60.2)	0.13	40.1 (34.7-43.7)	45.8 (36.6-52.6)	0.04

ETP_cmax _(mA/min)	119 (89-146)	109 (89-132)	0.13	145 (111-183)	123 (97-134)	0.003

ETP_AUC _(mA)	267 (167-350)	244 (161-294)	0.24	218 (87-307)	169 (76-268)	0.30

F1+2 (pmol/L)	202 (134-330)	301 (219-500)	0.88	307 (157-429)	471 (314-793)	0.78

TAT (μg/L)	5.3 (4.8-7.4)	16.2 (7.1-36.8)	< 0.001	7.9 (6.4-13.4)	27 (13.3-58.4)	< 0.001

D-dimers (μg/ml)	7.6 (2.1-21.2)	19.7 (4.4-37.6)	0.004	10.7 (3.1-34.8)	28.6 (7.3-51.4)	0.002

Values in median (interquartile range). Values during CVVH are medians of all samples. Postfilter values are actual values, not corrected for hemoconcentration.

aPTT = activated thromboplastin time; CVVH = continuous venovenous hemofiltration; ETP = endogenous thrombin potential; ETP_AUC _= area under the curve of the thrombin generation curve; ETP_Cmax _= maximal activity of ETP; ETP_Tlag _= ETP lag time = ETP_Tmax _= time to max ETP activity; F1+2 = prothrombin fragment; PTT = prothrombin time; SOFA = Sequential Organ Failure Assessment; TAT = thrombin-antithrombin complexes.

## Discussion

This randomized cross-over study in critically ill patients with AKI compared the hemostasis during anticoagulation with the LMWH nadroparin between two doses of CVVH using a cellulose tri-acetate filter. We found no signs of accumulation of anticoagulant activity in arterial blood and no signs of removal by filtration. Anticoagulant activity was quantified by anti-Xa activity. In arterial blood, anti-Xa levels peaked upon the intravenous nadroparin bolus and gradually declined thereafter despite the continuous infusion of the LMWH in the circuit, while postfilter anti-Xa activity remained constant. Anti-Xa activity was not detected in the ultrafiltrate.

It should be noted that we did not measure nadroparin concentration but its anticoagulant activity. If hemofiltration would remove the drug we would expect higher drug concentrations in group 1 with the lower CVVH, and assuming a linear relation between dose and effect, also a higher anti-Xa activity. The opposite was the case. Differences in anti-Xa activity between groups can therefore not be explained by a different handling of nadroparin by filtration. Another explanation is needed. Although the present study is of limited duration, a longer duration will likely not confer different results, because plasma anti-Xa activity did not tend to increase, it declined. Given the analytical precision of our test, relevant accumulation in plasma if present would have been detected. Corresponding to our findings, Joannidis and colleagues [13] found no accumulation of anti-Xa activity using the LMWH enoxaparin. The absence of removal of anticoagulant activity by filtration corresponds with a previous study [4], but not with a recent study [5]. The latter used a different LMWH (enoxaparin) and different membranes (polysulphone and acrylonitrile). LMWH are derived from unfractionated heparin by diverse ways of depolymerization, resulting in different mixtures with different molecular structures and features. Furthermore, Isla and colleagues [5] used membranes with a higher negative charge than the cellulose triacetate membrane used in our study [14]. Moreover, the sensitivity of our anti-Xa assay is sufficient to demonstrate relevant anti-Xa removal if present. Discrepancies between studies may therefore be related to the use of different types of LMWH and different membranes. Finally, nadroparin might also be removed by adsorption to the membrane. However, membranes are generally saturated after a couple of hours and accumulation would be expected thereafter. In addition, the present cellulose tri-acetate membrane has low adsorptive capacity. The absence of accumulation and removal, and the finding that the 2 L/h dose was not associated with higher anti-Xa activity indicates that nadroparin is cleared or inactivated in the body of these critically ill patients despite renal failure. This finding is striking because previous studies and a recent meta-analysis showed that renal insufficiency increases half-life of smaller heparin fragments causing accumulation of anti-Xa activity when glomerular filtration rate falls below 30 ml/min [2,3]. This seeming contradiction may be explained by other findings of this study.

Although arterial anti-Xa activity tended to decrease in time, postfilter anti-Xa activity was stable. Median postfilter anti-Xa activity was 1.7 times the arterial anti-Xa activity due to the extracorporeal administration of the LMWH. This finding corresponds to the results of Joannidis and colleagues [13]. It therefore seems rational to administer the LMWH in the extracorporeal circuit, especially because longer circuit life was associated with higher anti-Xa activities. However, other factors than nadroparin dose seem to influence anti-Xa activity and circuit life as well.

First, anti-Xa activity varied widely between patients. In addition, after correction for a difference in hemoconcentration, postfilter anti-Xa activity was higher in group 2 while nadroparin dose/blood flow ratio was lower. This discrepancy needs to be explained. Heparins mainly confer their anticoagulant effect by potentiating antithrombin, which primarily inhibits factor IIa and Xa. Heparin resistance may be due to low antithrombin concentrations. Supplementation of antithrombin to patients with low plasma concentrations does increase circuit life [15,16]. In our patients, baseline antithrombin correlated with anti-Xa activity. However, antithrombin was not lower in group 1, which had the lower anti-Xa activity, and antithrombin was not significantly lower in the patients with early filter clotting. Differences in anti-Xa activity between patients and groups may also be explained by the binding of heparin to proteins other than antithrombin, limiting the amount of heparin available to bind to antithrombin and thus decreasing the anticoagulant effect [17]. This heparin resistance was related to severity of disease: patients with high SOFA scores had lower anti-Xa activity. So called heparin-binding proteins are released from storage sites in endothelial cells [18]. Among these are acute-phase reactants such as platelet factor 4, histidine-rich glycoprotein, vitronectin, fibronectin and lipopolysaccharide-binding protein, which increase in sepsis [19,20]. Furthermore, heparin avidly binds to apoptotic and necrotic cells to discrete domains released from the nucleus into the membrane during apoptosis [21]. Apoptosis is a key mechanism in sepsis-related multi-organ failure [22]. Altogether, our finding that heparin resistance is related to the severity of disease has a strong pathophysiological base.

Some experiments demonstrate that LMWH binds less to plasma proteins than unfractionated heparin [19]. However, clinical studies report lower anti-Xa activity in response to LMWH in patients with deep vein thrombosis compared with young and elderly healthy volunteers [2], in critically ill patients compared with healthy volunteers [23], in intensive care patients, especially those with high body weight and multiple organ failure [24,25], and in critically ill patients on vasopressors [26]. The lower anti-Xa response in the above mentioned patients groups may be caused by non-specific binding of the LMWH to acute-phase proteins. Although our study is small, results are in accordance with those mentioned above. It suggests that the anticoagulant effects of LMWHs are inhibited in severely ill patients leading to anticoagulant failure, which goes undetected without anti-Xa monitoring. To optimize LMWH anticoagulation, monitoring of anti-Xa is therefore advocated in patients with high SOFA scores exhibiting early filter clotting.

We also aimed to explore whether ETP could have a role in monitoring systemic anticoagulation and circuit clotting. We found low baseline ETP compared with healthy volunteers. The pattern of ETP in arterial blood was opposite to anti-Xa activity with a strongly significant but weak correlation, likely reflecting LMWH anticoagulation. Postfilter ETP was lower than in arterial blood reflecting the extracorporeal administration of nadroparin. However, although postfilter anti-Xa activity was stable during CVVH, postfilter ETP increased in time. This indicates that ETP is not simply a marker of LMWH anticoagulation. Apparently, the capacity to generate thrombin gradually increased in postfilter blood despite 'adequate' anticoagulation. Increasing ETP in the hemoconcentrated blood leaving the filter likely reflects circuit-induced hypercoagulability due to a time dependent increase in procoagulant activity despite constant LMWH anticoagulant activity. This corresponds to the literature reporting that ETP is increased in various hypercoagulable states [6,27]. APTT and PTT reflect circulating coagulation factor concentrations, but do not reflect or predict an activated state of these factors or the ability to generate activated factors. ETP, by measuring plasma thrombin generation in time far beyond clot formation, is thought to fill that information gap. Finally, arterial ETP was inversely related to PTT, aPTT, TAT and SOFA score, suggesting that low ETP reflects consumption of coagulation factors due to increased thrombin generation as a result of critical illness. This assumption is supported by a clinical study reporting that ETP was lower in patients with overt disseminated intravascular coagulation [28] and by our observation that baseline ETP was lower in patients with early circuit clotting. Increasing postfilter ETP in time and lowered arterial ETP values with high TAT complexes may be two sides of the same coin, i.e. activation of coagulation factors in the extracorporeal circuit, causing a running coagulation cascade in the patient with net consumption of coagulation factors during increased thrombin formation. Altogether, our study confirms that ETP reflects interplay of the effects of low concentrations of coagulation factors due to consumption and heparin anticoagulation, both decreasing the capacity to form thrombin, and of extracorporeal hypercoagulability, which increases this capacity. Further studies are needed to determine which soluble factors cause this increased extracorporeal thrombin-generating capacity.

The present study further shows the complex relation between coagulation, anticoagulation, fibrinolysis, severity of disease and circuit clotting. Patients with early circuit clotting had longer PTT, aPTT and lower ETP. These prolonged coagulation times did not, however, protect against filter clotting. They were associated with early filter clotting indicating consumptive coagulopathy. Indeed, higher TAT complexes and D-dimers were also found, signaling higher prior thrombin generation. Most importantly, patients with early circuit clotting had higher SOFA scores. Short circuit life and high SOFA scores were additionally associated with lower levels of anti-Xa, despite a similar LMWH dose. Therefore, early circuit clotting in patients with high SOFA scores seems to be related to prior activation of coagulation with consumptive coagulopathy, heparin resistance and high extracorporeal thrombin generation.

Although our study is small and the results need to be confirmed, the finding of early filter clotting and heparin resistance in patients with severe organ failure corresponds to clinical experience and has a biochemical explanation. The finding suggests that heparins are not ideal for circuit anticoagulation in the most severely ill patients. In these patients regional anticoagulation with citrate may be preferred. In our recent randomized controlled trial in critically ill patients with acute renal failure comparing anticoagulation for CVVH with citrate to nadroparin anticoagulation, patient survival was better in those receiving citrate [29]. This difference was present in the entire group, but especially in the subgroups of patients with sepsis and higher SOFA score. Heparin resistance may be a second reason for not using heparins in the most severely ill patients.

## Conclusions

The present explorative randomized cross-over trial comparing hemostasis during anticoagulation with the LMWH nadroparin between two doses of CVVH showed no accumulation of anticoagulant activity and no signs of removal by filtration. However, the study suggests inactivation of the LMWH in patients with severe organ failure. Severe organ failure appeared as a major determinant of early circuit clotting due to prior systemic thrombin generation with consumptive coagulopathy, heparin resistance and elevated extracorporeal thrombin generation. In this setting the interpretation of ETP is complex, because it integrates the effects of low concentrations of coagulation factors due to prior thrombin generation and heparin anticoagulation, both decreasing the capacity to form thrombin, and extracorporeal activation of coagulation factors, which increases this capacity. Further studies are needed to define the role of ETP in monitoring circuit clotting.

## Key messages

• Anticoagulant activity of the LMWH nadroparin does not accumulate in patients with AKI receiving CVVH.

• The LMWH nadroparin is not removed by CVVH using a cellulose tri-acetate filter.

• LMWH seems to be inactivated in patients with severe organ failure.

• Severe organ failure seems a major determinant of early circuit clotting due to consumptive coagulopathy, heparin resistance and increased thrombin generation.

• The ETP integrates the effects of concentrations of coagulation factors, anticoagulation, prior thrombin generation and activation of coagulation factors on thrombin generation.

## Abbreviations

AKI: acute kidney injury; APACHE: Acute Physiology and Chronic Health Evaluation; aPTT: activated thromboplastin time; CVVH: continuous venovenous hemofiltration; ELISA: enzyme-linked immunosorbent assay; ETP_AUC_: area under the curve of the thrombin generation curve; ETP_Cmax_: maximal thrombin potential; ETP_Tlag_: time to start of thrombin generation; ETP_Tmax_: time to maximal thrombin generation; F1+2: prothrombin fragments 1 and 2; ICU: intensive care unit; IQR: interquartile range; LMWH: low molecular weight heparin; PTT: prothrombin time; RIFLE: Risk, Injury, Failure, Loss, End stage kidney; SAPS: Simplified Acute Physiology Score; SOFA: Sequential Organ Failure Assessment; TAT: thrombin-antithrombin complexes.

## Competing interests

The authors declare that they have no competing interests.

## Authors' contributions

HMO was involved in the concept and design of the study, in the analysis and interpretation of the data, and in the drafting and writing of the manuscript. MvS contributed to the biochemical measurements and the biochemical part of the database, the interpretation of the data and the writing of the manuscript. PJM performed the biochemical measurements and contributed to the interpretation of the data. JPJW contributed to the design of the study, the interpretation of the data and to the writing of the manuscript. AL contributed to the design of the study, in particular of the biochemical measurements, the interpretation of the data and the writing of the manuscript. All authors read and approved the final manuscript.
